# Hemodialysis Removes Uremic Toxins That Alter the Biological Actions of Endothelial Cells

**DOI:** 10.1371/journal.pone.0030975

**Published:** 2012-02-22

**Authors:** Kalliopi Zafeiropoulou, Theodora Bita, Apostolos Polykratis, Stella Karabina, John Vlachojannis, Panagiotis Katsoris

**Affiliations:** 1 Department of Biology, University of Patras, Patras, Achaia, Greece; 2 Department of Internal Medicine-Nephrology, University Hospital of Patras, Patras, Achaia, Greece; University of Arizona, United States of America

## Abstract

Chronic kidney disease is linked to systemic inflammation and to an increased risk of ischemic heart disease and atherosclerosis. Endothelial dysfunction associates with hypertension and vascular disease in the presence of chronic kidney disease but the mechanisms that regulate the activation of the endothelium at the early stages of the disease, before systemic inflammation is established remain obscure. In the present study we investigated the effect of serum derived from patients with chronic kidney disease either before or after hemodialysis on the activation of human endothelial cells *in vitro*, as an attempt to define the overall effect of uremic toxins at the early stages of endothelial dysfunction. Our results argue that uremic toxins alter the biological actions of endothelial cells and the remodelling of the extracellular matrix before signs of systemic inflammatory responses are observed. This study further elucidates the early events of endothelial dysfunction during toxic uremia conditions allowing more complete understanding of the molecular events as well as their sequence during progressive renal failure.

## Introduction

Chronic kidney disease (CKD) is due to a progressive loss of renal function that may lead to complications such as cardiovascular disease or pericarditis. To fully investigate the underlying cause of kidney damage, various forms of medical imaging, blood tests or renal biopsy are employed to find out if there is a reversible cause for the kidney malfunction. Established CKD or chronic renal failure (CRF) are terms that describe the late stage of kidney damage, when the disease is considered irreversible. Current therapy strategies aim the delay of the clinical manifestations to the final stages of the disease. Generally, agents targeting the functional control of the endothelium, such as angiotensin converting enzyme inhibitors (ACEIs) or angiotensin-II receptor antagonists (ARBs) are used, as they have been found to delay the clinical manifestations of the CKD [1], [2], [3], [4]. Nevertheless, even under treatment with ACEIs or ARBs, patients progressively lose proper renal function. For these reasons understanding the primary responses of the endothelium to each stages of the kidney disease is of major importance for the design of efficient therapeutic strategies.

The endothelium is the major site of control of vascular functions [5]. Under physiological conditions the vascular endothelium regulates processes that include vascular tone, vascular permeability to nutrients, macromolecules and leukocytes recruitment (and thus inflammation), platelet adhesion and aggregation, activation of the coagulation cascade and fibrinolysis [6], [7], [8], [9], [10], [11]. Endothelial dysfunction reflects the combination of altered endothelial properties resulting to improper preservation of organ function. Endothelial dysfunction might be characterized by altered basement membrane synthesis, increased vascular tone and permeability -which contributes to increased blood pressure and atherogenesis- and loss of antithrombotic and profibrinolytic properties. Such alterations do not necessarily occur simultaneously and may differ according to the nature of the injury and the intrinsic site-specific properties of endothelium.

CKD leads to altered properties and responses of the endothelium [12], [13], [14]. However, the mechanisms by which increased uremia might influence endothelial cells, and especially the early responses of endothelial cells to the stimuli present in the serum of patients with CKD, are still not well understood. Systemic exposure of the vasculature to uremic toxins may lead to endothelial activation [15] and to features associated with systemic inflammation like hypertension and atherosclerosis. Hemodialysis (HD) or nephrectomy are generally used as approaches for the treatment of the sort-term effects of renal disease [16], nevertheless patients under long term HD treatment do not have reduced risk of vascular disease [17], [18], [19], [20].

The present study was designed to investigate the short-term *in vitro* effect of serum from patients with CKD on endothelial cells and the relative effect of HD on endothelial cell activation and function. We focused on the early responses of the endothelial cells to the uremic toxins, before the inflammatory activation of the endothelial cells (expression of adhesion molecules, secretion of chemokines) is established. For this purpose sera from patients either before or after the HD procedure were collected and their relative effect on the activation of human umbilical vein endothelial cells was investigated. This experimental approach enabled us to clearly use the best internal controls available since the sera from the same patients were used. Our results clearly demonstrate that the initial response of endothelial cells to uremic toxins involves a rearrangement of the local micro-environment and extracellular matrix, a response that was up to date not appreciated.

## Materials and Methods

### Serum samples from CRF patients

Ten adult (men) patients on chronic maintenance HD, middle-aged 45±5 years old, who were clinically stable and free of active infection, autoimmune diseases or other traditional factors implicated to endothelial dysfunction (diabetes mellitus, hypertension, hyperlipidemia, smoking) and had no signs or symptoms of cardiovascular disease, participated in the study. None of the patients received antihypertensive drugs, immunosuppressive treatment, lipid-lowering agents, non-steroidal anti-inflammatory drugs or antioxidants such as vitamin E, C or allopurinol in the preceding 4 weeks. End stage kidney disease was attributed to glomerulonephritis in 3 cases, interstitial nephritis in 2 and polycystic kidney disease in 3 and was undetermined in 2 cases. The patients were routinely haemodialyzed three times weekly for 4.0 h with DCEA polysulfone membranes - surface 1.7 mm^2^, bicarbonate dialysate and low molecular weight heparin-enoxaparin as anticoagulation. The dialysate was endotoxin-free (Coatest Kabi Vitrum). Dialysis prescription was guided by the goal of achieving a value of Kt/V≥1.3. They were on erythropoietin therapy and the mean dosage was 90.5 (range 30.2–162) U/kg body weight/week. Body mass index (BMI) was calculated by dividing the weight in kilograms by the square of the height in meters.

For this study, we obtained ethics approval from the ethics committee of University of Patras.

### Endothelial cell culture

Primary human umbilical vein endothelial cells (HUVEC) were isolated from umbilical cord vein by collagenase digestion as previously described [21] and used at passages 2–4. The cells were grown as monolayers in M199 medium supplemented with 15% fetal bovine serum (FBS), 150 µg/ml endothelial cell growth supplement, 5 U/ml heparin sodium, 100 U/ml penicillin-streptomycin and 50 µg/ml gentamycin. Cultures were maintained at 37°C, 5% CO_2_ and 100% humidity.

### Migration assay

Migration assays were performed as previously described [22] in 24-well microchemotaxis chambers (Costar, Avon, France), using uncoated polycarbonate membranes with 8 µm pores. Briefly, HUVEC were harvested and resuspended at a concentration of 10^5^ cells/0.1 ml in medium containing 0.25% BSA. The bottom chamber was filled with 0.6 ml of medium containing 0.25% BSA and pre- or post-HD serum at dilutions ranging from 5% to 20% v/v. The upper chamber was loaded with 10^5^ cells and incubated for 4 h at 37°C. After completion of the incubation, the filters were fixed with saline-buffered formalin and stained with 0.33% toluidine blue solution. The cells that migrated through the filter were quantified by counting the entire area of each filter, using a grid and an Optech microscope at a 20× magnification.

### Cell proliferation assay

Cell number was assessed using the 3-[4,5-dimethylthiazol-2-yl]-2,5- dimethyltetrazolium bromide (MTT) assay [23]. HUVEC were seeded at 5×10^4^ cells/well in 24-well tissue culture plates in the corresponding culture medium. Cells were incubated in the absence of serum for 4 h. Pre- or post- HD serum was added to the medium of the cells at dilutions ranging from 5% to 20% v/v and the number of cells was measured after 48 h. MTT stock (5 mg/ml in PBS) at a volume equal to 1/10 of the medium was added and plates were incubated at 37°C for 2 h. The medium was then removed, the cells were washed with PBS pH 7.4 and 100 ml acidified isopropanol (0.33 ml HCl in 100 ml isopropanol) was added to all wells and agitated thoroughly to solubilize the dark blue formazan crystals. The solution was transferred to a 96-well plate and immediately read on a microplate reader (Biorad) at a wavelength of 490 nm.

Cell number was also determined by crystal violet assay: Adherent cells were fixed with methanol and stained with 0.5% crystal violet in 20% methanol for 20 min. After gentle rinsing with water, the retained dye was extracted with 30% acetic acid and the absorbance was measured at 590 nm.

### Annexin-V Binding Staining

The Annexin V-FITC Detection Kit I (PharMingen, San Diego, CA) was used according to the manufacturer's instructions. Cells were serum-starved for 4 h and pre- or post-HD serum was added to the medium of the cells at 20% v/v dilution. Cells were collected after 12 or 24 h incubation. Samples were analyzed in a FACScan flow cytometer (Becton Dickinson). For each sample, 10,000 ungated events were acquired.

### *In vitro* endothelial cell wound healing assay

HUVEC were grown in 6-well plates as confluent monolayers. The monolayers were incubated in the absence of serum for 4 h and wounded in a line across the well with a 200-µl standard pipette tip. Cells were washed twice with serum-free media and incubated with 20% v/v pre- or post-HD serum for 24 h. The area of the initial wound was photographed using a charge-coupled device camera connected to an inverted microscope (Axiovert 35; Zeiss, Thornwood, NY). The wound healing effect was calculated compared with the area of the initial wound.

### Gelatin Zymography

The activity of MMP-2 and MMP-9 was examined by zymography as previously described [24]. Endothelial cells were cultured in 6-well plates as confluent monolayer. HUVEC were serum-starved for 4 h and then incubated with culture medium supplemented with 20% pre- or post- HD serum. 4 h later the medium was replaced by minimal medium. 8 or 20 h later the media were collected, centrifuged, and aliquots of the supernatants were loaded with a non-reducing sample buffer onto a 10% sodium dodecyl sulfate (SDS)-polyacrylamide gel containing 1 mg/mL gelatin and electrophoresed. Gels were washed twice with 2.5% Triton X-100 solution for 30 min and once with 10 mM Tris-HCl buffer (pH 8.0) for 30 min. Gels were further incubated in 50 mM Tris-HCl (pH 8.0) containing 0.5 mM CaCl_2_ and 0.1 mM ZnCl_2_ at 37°C for 24 h. The gels were stained with 1% Coomassie Blue R-250 in 10% methanol and 5% acetic acid and subsequently destained with 10% methanol and 5% acetic acid. The relative amounts of MMP-9 and MMP-2 were quantified by NIH Image Analysis software and normalized to the total number of cells of each well (using Crystal violet method).

### Immunoblot analysis

Endothelial cells were cultured in 6-well plates as confluent monolayer. HUVEC were serum-starved for 4 h and then incubated with culture medium supplemented with 20% pre- or post- HD serum. 4 h later the medium was replaced by minimal medium. 8 or 20 h later the media were collected, centrifuged, and aliquots of the supernatants were analyzed by SDS-PAGE and proteins were blotted onto PVDF membranes (Millipore, Bedford, MA). Blocking was performed in a 5% fat-free dry milk in 0.2% Tween-20 in PBS and membranes were further incubated with primary antibodies for 1 h (1∶2000 dilution in TBS containing 0.1% Tween 20 (TBS-T) and 1% BSA), washed with 0.1% Tween- 20 in PBS, and then incubated with anti-mouse peroxidase-conjugated secondary antibody (1∶2000 in TBS containing 0.1% Tween 20 (TBS-T) and 3% fat free dry milk). Visualization of immunoreactive proteins was performed with enhanced chemiluminescence reagents (ECL kit; Amersham Pharmacia Biotech). The normalization was based on the number of cells of each well that was estimated using Crystal violet method.

### RNA isolation and reverse transcriptase-polymerase chain reaction analysis of MMP-2,-9 and TIMP-1, -2 mRNA

Total RNA was isolated from HUVEC cultured with 20% pre- or post-HD serum for 6, 12 or 24 h using Nucleospin® RNA II (Macherey-Nagel) according to manufacturer's instructions. Reverse transcriptase-PCRs were performed using the Access Reverse Transcriptase-PCR system (Promega). The sequences of the primers used in our studies are the following: GAPDH: 5′-TCT AGA CGG CAG GTC AGG TCC ACC-3′ and 5′-CCA CCC ATG GCA AAT TCC ATG GCA- 3′, MMP-2: 5′-ACA GTC CGC CAA ATG AAC C- 3′ and 5′-CCT GGG CAA CAA ATA TGA G- 3′, **MMP-9:** 5′-GCC TTG GAA GAT GAA TGG AA - 3′ and 5′- CAT CGT CAT CCA GTT TGG TG- 3′, **TIMP-1:** 5′-TGC AGT TTT CCA GCA ATG AG - 3′ and 5′-CTG TTG TTG CTG TGG CTG AT - 3′, **TIMP-2:** 5′-TTT GAG TTG CTT GCA GGA TG - 3′ and 5′-ATT TGA CCC AGA GTG GAA CG -3′, **COLLAGEN IV:** 5′-TTT CCA GGG TAG CCA GAT GCT C - 3′ and 5′-GGG GTT ACA AGG TGT CAT TGG G - 3′, **ELASTIN:** 5′-CCA TAC TTG GCT GCC TTA GC - 3′ and 5′-CAC TGG GGT ATC CCA TCA AG - 3′.

### Statistical analysis

Comparison of mean values among groups was done using ANOVA and the unpaired Student t-test. Homogeneity of variance was tested by Levene's test. Each experiment included at least triplicate measurements for each condition tested. All results are expressed as the mean ± SD of at least three independent experiments. Values of p less than 0.05 were taken to be significant (*p<0.05, **p<0.01, ***p<0.001).

## Results

### Uremic toxins present in the serum of patients with CKD alter endothelial cell properties *in vitro*

**Figure 1:**
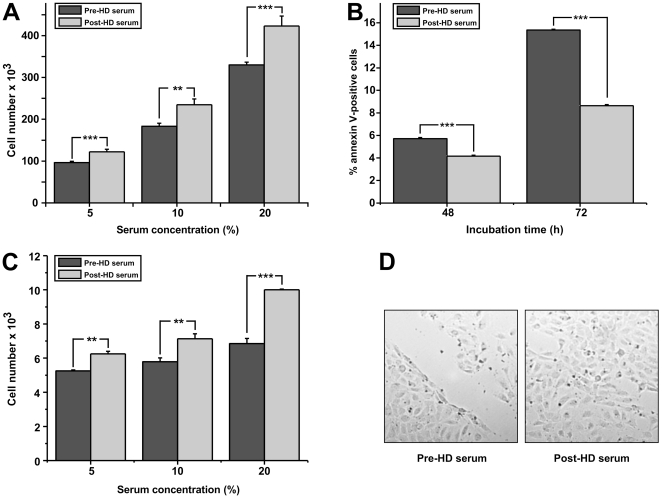
Effect of pre- or post-HD serum on proliferation, apoptosis, migration and wound healing activities of HUVEC. (A): HUVEC were incubated in medium supplemented with 5%, 10%, 20% pre- or post-HD serum and 48 h later their number was estimated by crystal violet. (B): HUVEC were incubated in medium supplemented with 20% pre- or post-HD serum and 48 or 72 h later the number of apoptotic cells was measured by FACS. (C): HUVEC were incubated in microchemotaxis chambers in culture medium supplemented with 5%, 10%, 20% pre- or post-HD serum and 4 h later the number of cells that migrated through the filter was quantified. (D) Endothelial monolayers were scratched, incubated in culture medium supplemented with 20% pre- or post-HD serum and 24 h later representative images of the plates were taken. Data are expressed as mean ± SEM of three independent experiments. ^*^, ^**^ and ^***^ represent p<0.05, p<0.01 and p<0.001 respectively.

Although CKD is positively associated with dysfunction of the endothelium, the effect of the uremic toxins of patients on the immediate responses of endothelial cells is not clearly demonstrated. To study the above mentioned effect, we isolated sera from patients with CKD right before or after HD, an approach that was not followed or appreciated in previous studies. At first we investigated the role of uremic toxins in the sera of our patients on the proliferation of endothelial cells. We incubated HUVEC with three different concentrations of sera. As shown in Figure 1A, incubation of HUVEC with increased concentrations of pre-HD serum resulted in reduced proliferation of endothelial cells compared to the relative concentrations of post-HD serum. Increased concentrations of serum resulted in increased proliferation in both pre- and post-HD sera, nevertheless in all the concentrations tested in our experiments the effect on proliferation was enhanced in the samples stimulated with post-HD serum. The stimulatory effect of the uremic toxins-free serum (post-HD serum) on the proliferation of HUVEC reached 30% compared to the pre-HD serum, when a concentration of 20% was used in the medium. These results indicate that the differential concentration mainly of uremic toxins present in the serum of patients with CKD before and after HD affects proliferation of primary endothelial cells in vitro.

Basal apoptosis can be observed in cultured cells under normal conditions. Furthermore, since post-HD serum is not believed to be completely free of uremic toxins, we investigated the effect of either pre- or post-HD serum on the apoptosis of HUVEC *in vitro*. We used the concentration of 20% of serum in the cultured medium where the effect on HUVEC proliferation was more pronounced. As shown in Figure 1B, incubation of endothelial cells with either pre- or post-HD sera leads to a time dependent increase in the percentage of apoptotic cells. This effect is more pronounced when cells are cultured with pre- HD serum and reaches an 80% increase in the number of apoptotic cells compared to cells cultures with post-HD sera after 72 hours. Nevertheless, the percentage of apoptotic cells in culture even under these conditions cannot be the soul reason for the decreased total number in our proliferation assays (Figure 1A), arguing for a combined effect of uremic-toxins on both the proliferation and cell survival of endothelial cells.

It is known that systemic inflammation affects the migration of endothelial cells, inhibiting the healing processes *in vivo* [25]. To study the effect of uremic toxins on migration, we incubated HUVEC with increasing concentrations of either pre-HD or post-HD serum. As shown in Figure 1C, pre-HD serum has a realtivelly small positive effect on the migration of endothelial cells in the different concentrations used. On the contrary, when post-HD serum was used, a dose-dependent induction on the migration of endothelial cells was observed that reached almost 70% induction at serum concentration of 20**%**.

The combination of endothelial cells proliferation/survival and migration *in vivo* might have an impact in wound healing processes. The effect of the concentration of uremic toxins before and after the HD in the healing responses of endothelial cells *in vitro* has not been so far addressed. To investigate this we used the widely used scratch assay. Wound repair was assessed 24 h after incubation with 20% pre- or post-HD serum. As shown in Figure 1D, endothelial wound repair in monolayers exposed to pre-HD serum was significantly lower than in cells exposed to post-HD serum.

### Uremic toxins present in the sera of patients with CKD promote the expression and activity of ECM-degrading proteinases

**Figure 2:**
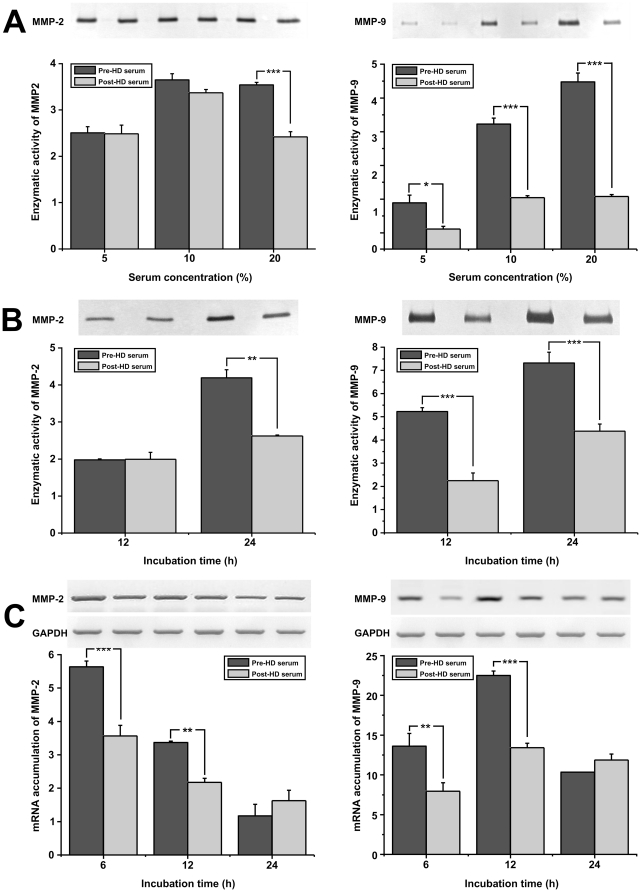
Effect of pre- or post-HD serum on MMP-2, -9 expression. (A): HUVEC were incubated in medium supplemented with 5%, 10%, 20% pre- or post- HD serum. 4 h later the medium was replaced by minimal medium and 20 h later the supernatants were analyzed for MMP-2 and MMP-9 activity by zymography. (B): HUVEC were incubated with culture medium supplemented with 20% pre- or post- HD serum. 4 h later the medium was replaced by minimal medium and 8 or 20 h later the supernatants were analyzed for MMP-2 and MMP-9 activity by zymography. (C): HUVEC were incubated in culture medium supplemented with 20% pre- or post- HD serum. 6, 12, or 24 h later total RNA was extracted from the cells, RT-PCR reactions were performed using specific primers for MMP-2, MMP-9 or GAPDH mRNAs, the PCR products were analyzed in agarose gels and quantified. Data are expressed as mean ± SEM of three independent experiments.. ^*^, ^**^ and ^***^ represent p<0.05, p<0.01 and p<0.001 respectively.

So far our results indicate that the uremic toxins alter the biological activities of endothelial cells *in vitro*, and are in accordance with previously published data [26], [27], [28], [29], [30]. Nevertheless, previous studies have been focused on the response of endothelial cell (*in vitro*) after long term stimulation with inflammatory stimuli or of the endothelium (*in vivo*) under conditions of systemic inflammation. We decided to focus on the early responses of endothelial cells upon stimulation with uremic toxins sera. At first we tested if the serum from the same patients before or after the HD could affect the deposition of extracellular matrix. We stimulated HUVEC with the above-mentioned sera and assessed for the activation of metalloproteinases (MMPs) either at the protein level by zymography on the medium of the cells or at the mRNA level by RT-PCR. As shown in Figure 2A, the pre-HD serum induces a dose dependent increase in both MMP-2 and MMP-9 protein levels in the medium of HUVEC as compared to the relevant samples where post-HD serum was used. Since the difference was more pronounced when 20% of either pre- or post-HD serum was added to the culture medium, we performed all following experiments using this serum concentration. As shown in Figure 2B, incubation of HUVEC with pre-HD serum led to increased MMP-9 activity in the cell supernatant after 12 and 24 hours, while it also increases MMP-2 protein levels after 24 hours as compared to the effect of the post-HD serum. Furthermore incubation of HUVEC with pre- or post- HD serum at the concentration of 20% induces MMP-2 and MMP-9 expression levels in a statistically significant manner reaching a maximum at 6 and 12 hours respectively (Figure 2C).

**Figure 3:**
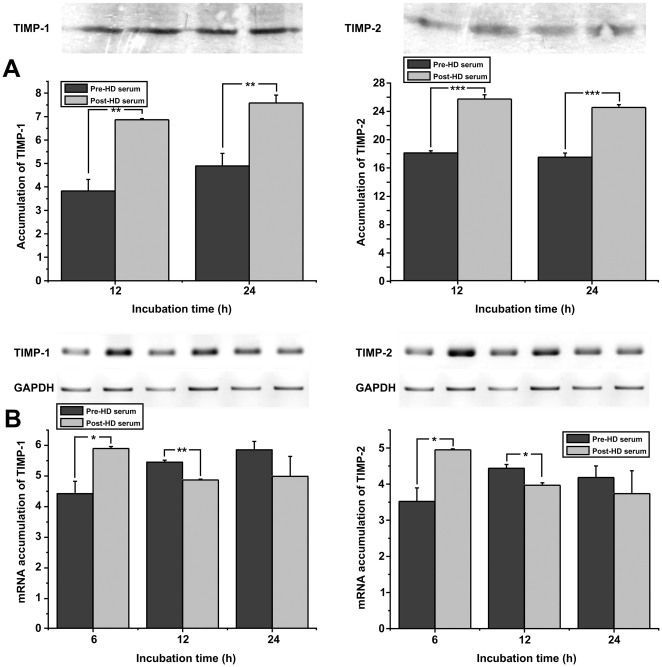
Effect of pre- or post-HD serum on the expression of TIMP-1 and TIMP-2. (A): HUVEC were incubated in culture medium supplemented with 20% pre- or post- HD serum. 4 h later the medium was replaced by minimal medium and 8 h or 20 h later the supernatants were analyzed for TIMP-1 and TIMP-2 proteins by SDS-PAGE. (B): HUVEC were incubated with culture medium supplemented with 20% pre- or post- HD serum. 6, 12, or 24 h later total RNA was extracted from the cells, RT-PCR reactions were performed using specific primers for TIMP-1, TIMP-2 or GAPDH mRNAs, the PCR products were analyzed in agarose gels and quantified. Data are expressed as mean ± SEM of three independent experiments. ^*^ and ^**^ represent p<0.05 and p<0.01 respectively.

Since both *in vitro* and *in vivo*, activity of MMPs is counter-balanced by the expression of naturally expressed inhibitors (tissue inhibitors of metalloproteinases, TIMPs), we assessed the expression levels of TIMP-1 and TIMP-2 protein levels on supernatants from HUVEC that were stimulated with sera at the concentration of 20%. As shown in Figure 3A, we observed a statistically significant increase of TIMP-1 and TIMP-2 protein levels when HUVEC were incubated with post-HD serum, compared to pre-HD serum. This increased accumulation of TIMP-1 and -2 proteins in the medium of cells cultured with uremic-free sera can be attributed to increased expression of the relative genes, as shown in Figure 3B.

### Uremic toxins present in the sera of patients with CKD inhibit the expression of extracellular matrix proteins

**Figure 4:**
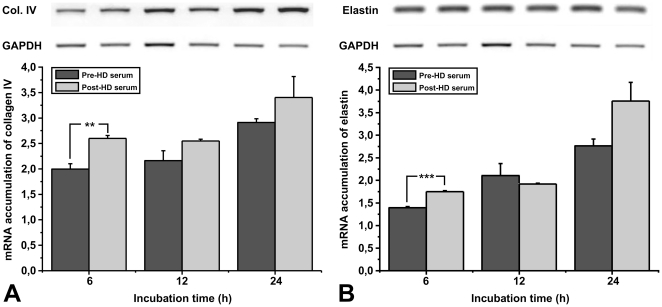
Effect of pre- or post-HD serum on the expression of collagen-IV and elastin. HUVEC were incubated with culture medium supplemented with 20% pre- or post- HD serum. 6, 12, or 24 h later total RNA was extracted from the cells, RT-PCR reactions were performed using specific primers for Collagen IV, Elastin or GAPDH mRNAs, the PCR products were analyzed in agarose gels and quantified. Data are expressed as mean ± SEM of three independent experiments. ^*^, ^**^ and ^***^ represent p<0.05, p<0.01 and p<0.001 respectively.

To investigate the effect of uremic toxins in the sera from patients with CKD on the expression of extracellular matrix components by endothelial cells we analyzed the expression levels of collagen IV and elastin. As shown in Figure 4, incubation of endothelial cells with either pre- or post-HD sera induces the expression of both collagen IV and elastin. This increase is more pronounced when endothelial cells are incubated with the post-HD serum. These data suggest that uremic toxins could inhibit the immediate healing response of endothelial cells *in vivo* after injury or local endothelial loss due to apoptosis.

## Discussion

Uremic toxins contribute to endothelial dysfunction in CKD. In renal failure, endothelial dysfunction and cardiovascular complications are closely linked [14], [31]–[35]. Endothelial cell damage correlates with thrombosis, hypertension and may be also responsible for accelerated atherosclerosis in patients with CKD. Traditional risk factors cannot explain the high incidence of cardiovascular disease in patients with CKD therefore several studies focus on other parameters such as endothelial dysfunction or insulin resistance. In this paper, we focused on the mechanism of endothelial dysfunction induced by uremic toxins, including the remodelling of the extracellular matrix.

Our results demonstrate that uremic toxins modulate primary responses of endothelial cells that correlate with the function of endothelium *in vivo*. Endothelial loss, due to apoptosis leads to vascular complications like thrombosis, increased inflammatory infiltration to the intimal area of the arteries and impaired NO production that lead to hypertension and atherosclerosis. Our results demonstrate that uremic toxins induce apoptosis of endothelial cells *in vitro*. This result is in line with previous reports that link increased endothelial cell apoptosis and renal failure, an effect that was mainly attributed to increased levels of AGEs [34], [36]. *In vivo* loss of the endothelial monolayer leads to the formation of a thrombus but it might also be compensated by the increased proliferation of endothelial cells neighboring the damaged area or from direct recruitment of endothelial cell precursors from the bone marrow or circulation [37]. Our results indicate that in parallel to the increased apoptosis that is observed in endothelial cells when they are incubated with media containing uremic toxins, a decreased proliferation and migration capacity of the endothelial cells is observed. These results might also explain the thrombophilia that is observed in patients with renal failure that often leads to vascular clotting, heart attacks and strokes. The fact that increased apoptosis, reduced proliferation and inhibition of migration were dependent on the concentration of the serum as well as the fact these effects were reversed by HD, indicate that the primary response of endothelial cells to ingredients of the sera in patients with CKD is to the uremic toxins. This result is of particular interest since in a previous study low levels of AGEs were not linked to a better survival rate in HD patients [38]. Our results indicate thought that HD significantly improves endothelial biological functions *in vitro* indicating that the primary endothelial damage is related most probably to uremic toxins.

Several studies indicated the role of disturbed extracellular matrix metabolism in defective myocardial and vascular remodelling [39]–[52]. Therefore MMPs as well as their tissue inhibitors have been proposed as a group of factors that add to the pathogenesis of atherosclerosis. Animal models and *in vitro* investigations have shown their multifaceted actions, varying from protective and anti-atherogenic in the case of TIMP-2, through neutral of TIMP-1 or ambiguous of MMP-9, to pro-atherogenic of MMP-2 [52]–[57]. Moreover, gelatinases A and B (MMP-2 and MMP-9) constitute risk factors for myocardial infarction [58] whereas their tissue inhibitors TIMPs have an impact on postmyocardial infarction remodelling [59], [60] and correlate positively with left ventricular mass and wall thickness [61]. The role of MMPs in CKD has been studied extensively [62]–[65], and especially the impact on ischemic acute renal injury and scarring in the course of glomerulopathies [66]–[68].

Our data demonstrate that uremic toxins modulate the expression of MMP production by endothelial cells *in vitro* as well as their enzymatic activity. Interestingly, our data clearly demonstrate an opposite effect of uremic toxins on the production of MMPs and TIMPs. When the protein levels were evaluated a clear reduction in the production of TIMP-1 and -2 was observed in endothelial cells that were cultured with pre-HD serum. Relative expression levels of TIMP RNA supported these results. The initial strong induction of TIMP-1 and -2 expression levels when endothelial cells were cultured with post-HD serum were followed by a moderate increased production of TIMPs at prolonged periods of incubation of endothelial cells with pre-HD serum. This response of the cells to uremic sera can only be explained by a time-dependent and well-orchestrated response of endothelial cells in “danger signals” *in vivo* [69]–[72]. The original response of endothelial cells to uremic toxins leads to the activation of MMPs without affecting the initial levels of TIMPs. Further exposure of the endothelium might initiate the activation of inhibitory pathways that aim the resolution of the inflammation produced by the uremic toxins. In the absence of obvious uremia (like in the case of post-HD serum) the relative production of MMPs versus TIMPs seems to be more balanced which most probably reflects the response of a functional endothelium *in vivo*. Such an endothelial response to uremic toxins *in vivo* might lead to degradation of the extracellular matrix and the basal membrane of the endothelial cells, leading to increased endothelial loss due to anoikis *in vivo*. One could speculate that the expression of MMPs might potentiate the ability of the endothelial cells to modulate the extracellular matrix locally and induce wound healing. Though, this is most probably not the case since our experiments clearly demonstrate a decreased ability of endothelial cells to migrate and cover damaged regions *in vitro*. The increased MMP production seems to correlate more with the increased thrombotic incidence and atherosclerosis observed in patients with kidney disease. Increased MMP activity correlates with atherosclerosis development and plaque rupture. Plaque vulnerability especially is linked to intimal thickening, loss of the collagen fibrous cap surrounding mostly necrotic areas of atherosclerotic lesions, or increased angiogenesis and hemorrhage within the atherosclerotic lesions. Our results suggest that uremic toxins, by modulating the expression and activity of MMPs might be major regulators of the thrombogenic incidents observed in patients with CKD.

Our results demonstrate that endothelial cells contribute at the early response to uremic toxins to the overall systemic inflammation observed in patients with CKD. This response resembles the procedure of the activation of the endothelium during atherosclerosis. During the early stages of atherosclerosis progression endothelial cells express adhesion molecules (e.g. VCAM-1, ICAM-1, E-selectin) as well as chemokines (e.g. MCP-1, MCP-3, Fractalkine) [25], [69], [73], [74] upon activation of the nuclear factor-kappa B [75]. This response leads to the recruitment of immune cells (namely monocytes) into the intima of the large arteries. At latter stages the disease does not depend on the endothelial response but mainly to the cells of the immune system either resolve the initial inflammation or activate an adaptive immune response [76]–[78]. We would like to propose a similar process during the development and establishment of CKD At the early stages the accumulation of uremic toxins in the plasma of patients could lead to altered functions of the endothelium, reorganization of the extracellular matrix of the large arteries and an inflammatory response. At latter stages, the presence of uremic toxins might have no effect on the activation of specific molecular response in endothelial cells, though it could contribute to increased apoptosis and reduced wound healing. The practical significance of this hypothesis, and the stage where the progression of the disease is still endothelial-dependent definitively deserves further investigation.

The advantage of this study relies on the fact that we used pre-HD and post-HD sera from the same patients. To our understanding our approach provided the best internal controls to study the direct effect of uremic toxins present in patients with CKD on endothelial cells. This approach prevented us from using sera from healthy patients since HD could not be applied. Nevertheless this was not the aim of this study that aimed on the net effect or uremic toxins on endothelial cells and not the overall effect of sera from patients with CKD compared to normal sera on endothelial cells (an approach that was used extensively in the past). Our results demonstrate that uremic toxins contribute to a great extend to deregulation of endothelial cells, most probably at the early stages of the development of kidney disease. Further studies are ongoing regarding the clinical evaluation and significance of our results.
